# Self-Driving Car Location Estimation Based on a Particle-Aided Unscented Kalman Filter

**DOI:** 10.3390/s20092544

**Published:** 2020-04-29

**Authors:** Ming Lin, Jaewoo Yoon, Byeongwoo Kim

**Affiliations:** Department of Electrical Engineering, University of Ulsan, 93 Daehak-ro, Nam-gu, Ulsan 44610, Korea; flaaud159@naver.com (M.L.); jaewoo127@naver.com (J.Y.)

**Keywords:** particle filter, sensor fusion, self-driving car, unscented Kalman filter, vehicle model, Monte Carlo localization

## Abstract

Localization is one of the key components in the operation of self-driving cars. Owing to the noisy global positioning system (GPS) signal and multipath routing in urban environments, a novel, practical approach is needed. In this study, a sensor fusion approach for self-driving cars was developed. To localize the vehicle position, we propose a particle-aided unscented Kalman filter (PAUKF) algorithm. The unscented Kalman filter updates the vehicle state, which includes the vehicle motion model and non-Gaussian noise affection. The particle filter provides additional updated position measurement information based on an onboard sensor and a high definition (HD) map. The simulations showed that our method achieves better precision and comparable stability in localization performance compared to previous approaches.

## 1. Introduction

In recent years, research on self-driving cars has gained much prominence. The ultimate goal of self-driving cars is to transport people from one place to another without any help from a driver. A self-driving system must control numerous parameters, including speed, orientation, acceleration, and maneuvering, in order to drive without any human assistance. All of these control parameters are controlled by the decision-making module, which handles all perception data from the vehicle and sensors. The perception module determines the relationship between the ego vehicle and the surrounding environment. One of the most important algorithm modules is vehicle localization because all the sensors sense the environment based on local vehicle coordinates [1]. The typical perception sensors of a self-driving car are the camera, radar, light detection and ranging (LIDAR), 2D laser scanner, global positioning system (GPS), and inertial measurement unit (IMU) [2]. GPS is the most commonly used navigation system in self-driving cars. However, because of issues with multipath routing and poor signal availability in cities, relying entirely on GPS is not suitable for localizing vehicles in urban environments. Although differential GPS systems can be used, the high cost and size of these systems limit their implementation [3]. Furthermore, GPS systems cannot be used in tunnels or indoor environments. Vision-based localization has been proposed as a method for localizing vehicles using a low-cost camera. However, the vision-based localization algorithm is easily affected by weather and light conditions, leading to insufficient accuracy and stability [4,5,6,7,8]. LIDAR-based map matching can yield highly precise results with the help of a high definition (HD) map. By contrast, matching requires the environment to be accurately mapped such that the point cloud of the environment does not change. Point cloud matching is expensive and requires considerable power and computation resources [9,10,11,12,13,14,15,16]. Thus, developing a low-cost localization method that can utilize the vehicle’s sensors and road infrastructure to achieve precise and stable location performance is necessary for furthering research on self-driving cars. One of the localization solutions that can be used in complex urban environments is vehicle localization based on local sensor systems and information from the HD map. Matching an entire point cloud with an HD map is inefficient; therefore, only the ground truth location map of infrastructures is used in this study, for computational efficiency. In previous research, vehicle localization based on vehicle-to-vehicle (V2V) and vehicular ad-hoc network (VANET) communication was proposed. The basic condition is that these algorithms require surrounding vehicles to be equipped with V2V communication equipment, which are then referred to for the infrastructures [17,18,19,20,21,22,23]. In this study, we only consider the case of a single vehicle. Moreover, because the vehicle data contain a large amount of noise, an efficient filtering algorithm is needed to obtain precise localization results.

The methods for vehicle localization have improved considerably over the years. The primary methodology that was used is the probabilistic approach. The Kalman filter (KF) is an optimal estimator that is designed for processing Gaussian noise with mean and variance, and it is an important component in several such approaches [24]. One of the assumptions of the KF is that the noise should be Gaussian. However, in practice, a function like the trigonometric filter renders the Gaussian noise non-Gaussian. Therefore, an extended Kalman filter (EKF), which uses a low order Taylor expansion to linearize the nonlinear (e.g., trigonometric) function, has been proposed. It uses a partial derivative to represent the rate of change of the nonlinear functions, which aims to keep the noise Gaussian. If the state is a vector, then the partial derivative parameters can be assembled into a new matrix, which is called a Jacobian matrix. Generally, in order to localize the vehicle’s position, researchers derive the Jacobian matrix based on the transition and measurement models for handling the vehicle’s noisy sensor data [25,26,27,28,29,30]. If an EKF based on a Jacobian matrix approximates a nonlinear function using a high order of Taylor series, it also works well in transforming nonlinear functions into linear ones. The critical problem, however, is that the Jacobian matrix is difficult to derive for complex dynamics. Therefore, a new, sample region-based Kalman filter, which is called the unscented Kalman filter (UKF), was proposed. The UKF performs better than the EKF and KF when the system model is highly nonlinear [31,32,33]. The UKF uses some key points, which are called sigma points, to approximate the non-Gaussian noise into Gaussian based on the unscented transform. In this way, it can properly capture the nonlinearity. Furthermore, because the UKF approximates the non-Gaussian noise with sigma points, it is easy to combine other information when selecting the sigma points and there is no need to calculate the Jacobian matrix.

The basic assumption of the Kalman filter family is that noise is Gaussian. In the real world, most noise does not have a Gaussian property. For processing non-Gaussian noise, a Monte Carlo-based localization approach, called particle filter (PF), has been proposed [34,35]. The particle filter uses several samples, referred to as particles, to approximate the non-Gaussian property. Because the particles are generated randomly, they can represent the properties of non-Gaussian noise precisely if there are sufficient numbers. However, a vehicle has limited computational resources; therefore, it cannot allow the particle filter to approximate the number of particles. Therefore, there is a trade-off between precision and computational resources when generating an effective particle-based system model. Thus, an extended Kalman filter-aided particle filter, called an extended particle filter (EPF), and an unscented particle filter (UPF), called the Kalman filter-aided particle filter, have been proposed [36,37,38]. Both the EPF and the UPF use system models to generate and update the particles. It should be noted that each particle should compute the sigma points or Jacobian matrix; therefore, both the EPF and UPF are computationally inefficient and difficult to implement [39,40,41].

In this study, we propose a new method, the particle-aided unscented Kalman filter (PAUKF), for vehicle localization. With the help of the particle filter, the unscented Kalman filter can estimate a system with high nonlinearity and various sources of nonlinear noise more precisely. Because each particle does not have to update the sigma points or share the prediction model, this method requires fewer computational resources. The computational burden and precision of PAUKF can be easily tuned by tuning the quantity of the sigma points and particles. The results of the simulation show that the PAUKF estimates the vehicle’s position and state more accurately than other methods that use a limited number of particles. Section 2 illustrates the methodology of the PAUKF. Section 3 details the simulation conditions, and Section 4 presents the analysis of the simulation results. Finally, Section 5 presents the conclusion of this paper.

## 2. Particle-Aided Unscented Kalman Filter

This section describes the implementation of the PAUKF, including particle implementation and PAUKF implementation. Both the PF and UKF are Bayesian-based filters, and the environment is assumed to be Markov, which means that the PAUKF also has a Markov assumption.

### 2.1. Particle Filter Algorithm

**Figure 1:**
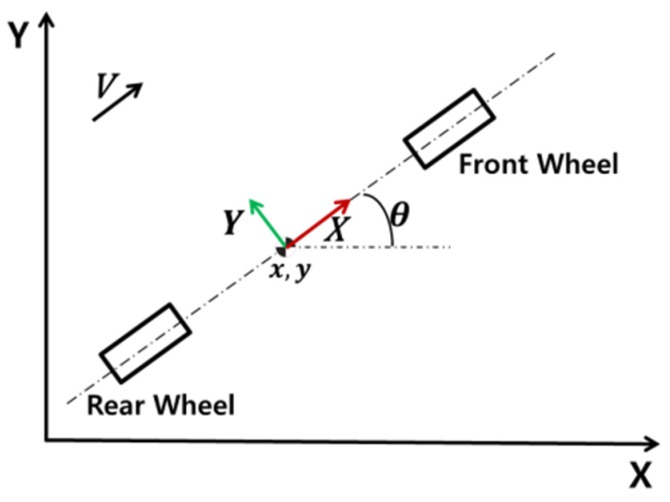
Vehicle state in two-dimensional Cartesian space.

The particle filter is a Monte Carlo-based method that can handle both Gaussian noise and non-Gaussian noise [42]. Because the vertical movement of the vehicle is small, we only consider the vehicle in a two-dimensional Cartesian space with the vehicle heading θ. A bicycle model is used in this study to represent the motion of the vehicle because a complex vehicle model aggravates the computational burden, and many parameters cause additional noise [43]. The state of the vehicle is represented by <x, y, and θ>, as shown in Figure 1.

The inputs that we use are the range sensor and the ground truth of the infrastructures in the HD map. The final position of the vehicle should be the best posterior belief based on past data and the current state. The particle filter is a nonparametric implementation of the Bayes filter, which uses a finite number of samples to approximate the posterior. Thus, the final belief bel(x) should be generated for each particle by using each important factor (weight), as shown in Equation (1). The $x_{\left[1,2\ldots N\right]}$ means the state vector of each particle and $w_{\left[1,2\ldots N\right]}$ is the weight of each particle. The size of each particle X was 3 × 1. W is a non-negative factor termed as the importance factor. In this study, we used 100 particles for simulation, which means that N is 100. The larger the importance factor, the more it affects the final estimation result.

(1) $$\mathrm{bel}\left(x\right)=\sum_{i=1}^{N}x_{N}w_{N}.$$

The particle filter for localization can be divided into four parts, which are introduced in the following subsections.

#### 2.1.1. Initialization

To localize the vehicle in global coordinates, it is essential to provide an initial location to the vehicle. Otherwise, the vehicle will search for its position over the entire world. Therefore, we use the GPS sensor for initialization. Even though the GPS signal is poor due to multipath and blocking issues, it still provides a limited area for the vehicle to localize. Because the particle filter is recursive, after initialization, the noisy GPS signal data are filtered recursively. When a particle filter receives GPS data, it generates N random particles for initialization.

#### 2.1.2. Prediction

To obtain the prior belief, each particle at timestamp k − 1 should predict the current state based on the system prediction model. The prediction model was constructed based on the vehicle model. Complex models, such as a dynamic model with tires, can also be included. However, complex vehicle models reduce computation efficiency. Such models also require detailed vehicle parameters, which are difficult to set. Incorrect parameters can cause noisy estimations. Considering the computational burden and precision, a kinematic model was used in this study [43]. We ignore the slip angle because vehicle travel in cities is typically not fast.

(2) $${\bar{X}}_{k+1}={\left[\begin{array}{c} \bar{x}\\ \bar{y}\\ \bar{\theta } \end{array}\right]}_{k+1}=[\begin{array}{c} \frac{v_{k}}{\dot{\theta_{k}(\Delta t)}}[\sin(\theta_{k}+\dot{\theta_{k}(\Delta t)})-\sin(\theta_{k})]\\ \frac{v_{k}}{\dot{\theta_{k}(\Delta t)}}[\cos(\theta_{k})-\cos(\theta_{k}+\dot{\theta_{k}(\Delta t)})]\\ \dot{\theta_{k}(\Delta t)} \end{array}]+{\left[\begin{array}{c} x\\ x\\ \theta \end{array}\right]}_{k}.$$

As Equation (2) shows, the prediction contains several trigonometric functions, which correspond to a highly nonlinear prediction model. The theta angle of each particle is critical because it changes according to the local vehicle coordinates. The kinematic model incorporates several assumptions such as the value of $\dot{\theta }$ being equal to zero.

#### 2.1.3. Weight Calculation

Weight is also an important factor that can heavily influence particle motion. Measurements from the range sensor were used to calculate the weight. We assume that the vehicle can receive all the data from the vehicle-to-everything (V2X), HD map, and range sensor. Thus, the vehicle can receive the distance data and orientation between the vehicle and every exterior infrastructure. Here, $d_{i}$ is the measured distance between the vehicle and infrastructure i, $\epsilon_{d}$ is the distance measurement noise, and Δθ is the relative orientation angle of the vehicle and infrastructure i. As Equations (3) and (4) show, the measurement model is nonlinear in nature.

(3) $$Z_{k+1\;}=\;f({\bar{X}}_{k+1})+{\mathrm{Noise}}_{k+1},$$

(4) $$Z_{k+1}={\left[\begin{array}{c} d_{\left[i\right]}\\ \Delta \theta_{\left[i\right]} \end{array}\right]}_{k+1}={\left[\begin{array}{c} \sqrt{{\left({\bar{x}}_{k}-x_{b,i}\right)}^{2}+{\left({\bar{y}}_{k}-y_{b,i}\right)}^{2}}\\ \arctan\left(\frac{{\bar{x}}_{k}-x_{b,i}}{{\bar{y}}_{k}-y_{b,i}}\right) \end{array}\right]}_{k+1}+{\left[\begin{array}{c} \epsilon_{d}\\ -{\;\theta }_{v}+\epsilon_{\Delta \theta } \end{array}\right]}_{k+1}.$$

To evaluate the weight, a multivariable normal distribution function was used to assess the importance of each particle. Thus, a multivariable normal distribution function returns the weight of a particle based on the newest sensor measurement values and the predicted values from the model as

(5) $$w_{i}=\;p\left(x_{i},y_{i}\right)=\frac{1}{2{\pi \sigma }_{x}\sigma_{y}}e^{-\left(\frac{{\left(x-\mu_{x}\right)}^{2}}{2\sigma_{x}^{2}}+\frac{{\left(y-\mu_{y}\right)}^{2}}{2\sigma_{y}^{2}}\right)}\;i\;=1,2\ldots N.$$

#### 2.1.4. Resampling

After calculating the weight and prediction values, the particle filter should select the particle along with its corresponding weight, which is the resampling step.

The entire PF part of the algorithm’s pseudo code is shown in Table 1.

### 2.2. Particle-Aided Unscented Kalman Filter Algorithm

**Figure 2:**
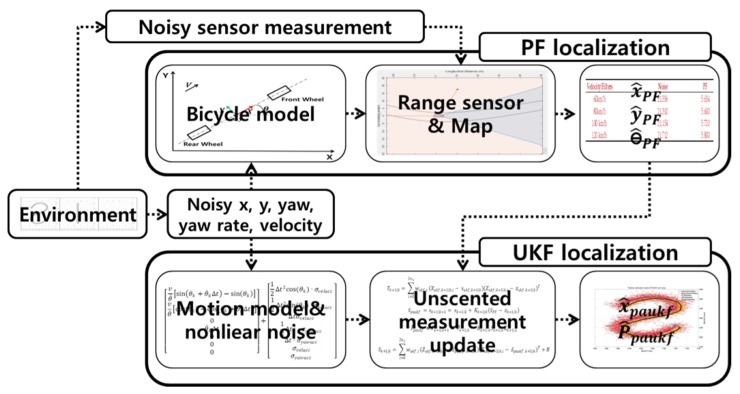
Particle-aided unscented Kalman filter algorithm flowchart.

The particle filter algorithm is introduced in Section 2.1. The particle estimates the position of the vehicle by using the range sensor. The final results for each particle contain information about the surrounding infrastructures and the position of the ego vehicle. Therefore, it can be concluded that this sensor provides more accurate results. When the UKF estimates the state, the results from the particle filter will be the measurement value of the vehicle. Subsequently, the PAUKF can extract a more precise result based on the particle filter estimation results. A flowchart of the PAUKF is shown in Figure 2.

The UKF is a Bayesian filter that has better performance than the EKF when estimating the state of a discrete-time nonlinear dynamic system. Because it is based on a Kalman filter, the framework of a UKF is almost the same as that of a KF. The difference is that a UKF performs stochastic linearization by using the weighted statistical linear regression process, known as an unscented transform. Instead of using a Taylor expansion, a UKF deterministically extracts the mean and covariance using the sigma points.

The sigma points are predefined by an empirical parameter λ that is calculated using Equation (6). The sigma point is a symmetrical region around the mean value. $P_{k|k}$ is the covariance matrix of the state, which updates at every iteration. The state vector of the vehicle is $x_{k}$, which is a 5 × 1 vector, as shown in Equation (7). The state vector value is the mean of the sigma matrix. $n_{x}$ is the quantity of the state vector with a size of 5. Then, the sigma points are generated using Equation (8).

(6) $$\lambda \;=3-{\;n}_{x},$$

(7) $$x_{\mathrm{paukf},\;k}={\left[\begin{array}{ccccc} x & y & v & \theta & \dot{\theta } \end{array}\right]}^{T},$$

(8) $$X_{\mathrm{paukf},\;k}=\left(\mu_{k},{\;\mu }_{k}+\sqrt{\left(\lambda \;+{\;n}_{x}\right)P_{k}},{\;\mu }_{k\;}-\sqrt{\left(\lambda \;+{\;n}_{x}\right)P_{k}}\right).$$

After it generates the sigma points, a UKF needs a prediction model to determine the prior probability of the state. To obtain a more precise position regarding position, a UKF considers more states of the vehicle and the effect of nonlinear noise on the states.

The constant turn rate and velocity magnitude (CTRV) vehicle motion model was used in this study [44]. Based on the CTRV model, the discrete state transition model is derived by integrating the differential equation of the state, which considers the nonlinear process noise vector. Considering the underlying structure of the vehicle for a real-world test, the acceleration and yaw acceleration are considered to be noise. In particular, the acceleration and yaw acceleration noise effects are nonlinear. Therefore, the process noise cannot be handled by addition alone. In order to handle nonlinear noise, it is considered to be a state, as shown in Equation (9). This means that the size of $x_{\mathrm{ukf}}$ becomes $x_{k,\;\mathrm{aug}}$, which is a 7 × 1 vector.

(9) $$x_{\mathrm{paukf},\;k,\mathrm{aug}}={\left[\begin{array}{ccccccc} x & y & v & \theta & \dot{\theta } & w_{\mathrm{velacc}} & w_{\mathrm{yawacc}} \end{array}\right]}^{T}.$$

The process noises $w_{\mathrm{velacc}}$ and $w_{\mathrm{yawacc}}$ are set as normal Gaussian distributions with variances of ${\;\sigma }_{\mathrm{velacc}}{}^{2}$ and $\sigma_{\mathrm{yawacc}}{}^{2}$, respectively, as shown in Equations (10) and (11).

(10) $$w_{\mathrm{velacc}}\;\sim \;N\left(0,{\;\sigma }_{\mathrm{velacc}}{}^{2}\right),$$

(11) $$w_{\mathrm{yawacc}\;}\sim \;N\left(0,{\;\sigma }_{\mathrm{yawacc}}{}^{2}\right).$$

The covariance matrix $P_{k}$ is also augmented into $P_{k,\mathrm{aug}}$, which has a size of 7 × 7, as shown in Equation (12).

(12) $$P_{k,\mathrm{aug}}=\left[\begin{array}{ccc} P_{k} & 0 & 0\\ 0 & \sigma_{\mathrm{velacc}}{}^{2} & 0\\ 0 & 0 & \sigma_{\mathrm{yawacc}}{}^{2} \end{array}\right].$$

The process model is derived based on the CTRV assumption and noise, as shown in Equation (13). The model that we derive has highly nonlinear properties, as indicated in Equation (14).

(13) $$x_{\mathrm{paukf},k+1}={\;x}_{\mathrm{paukf},k}+\int_{k}^{k+1}{\dot{x}}_{\mathrm{paukf},k}\mathrm{dt},$$

(14) $$x_{\mathrm{paukf},k+1,\mathrm{aug}}=f(x_{\mathrm{paukf},k,\mathrm{aug}},\sigma_{\mathrm{velacc}},\sigma_{\mathrm{yawacc}})=x_{\mathrm{paukf},k,\mathrm{aug}}+[\begin{array}{c} \frac{v}{\dot{\theta }}[\sin(\theta_{k}+{\dot{\theta }}_{k}\Delta t)-\sin(\theta_{k})]\\ \frac{v}{\dot{\theta }}[\cos(\theta_{k})-\cos(\theta_{k}+{\dot{\theta }}_{k}\Delta t)]\\ 0\\ {\dot{\theta }}_{k}\Delta t\\ 0\\ 0\\ 0 \end{array}]+\;[\begin{array}{c} \frac{v}{\dot{\theta }}[\sin(\theta_{k}+{\dot{\theta }}_{k}\Delta t)-\sin(\theta_{k})]\\ \frac{v}{\dot{\theta }}[\cos(\theta_{k})-\cos(\theta_{k}+{\dot{\theta }}_{k}\Delta t)]\\ 0\\ {\dot{\theta }}_{k}\Delta t\\ 0\\ 0\\ 0 \end{array}]+[\begin{array}{c} \frac{1}{2}\Delta t^{2}\cos(\theta_{k})\cdot \sigma_{\mathrm{velacc}}\\ \frac{1}{2}\Delta t^{2}\sin(\theta_{k})\cdot \sigma_{\mathrm{velacc}}\\ \Delta t\sigma_{\mathrm{velacc}}\\ \frac{1}{2}\Delta t^{2}\cdot \sigma_{\mathrm{yawacc}}\\ \Delta t\cdot \sigma_{\mathrm{yawacc}}\\ \sigma_{\mathrm{velacc}}\\ \sigma_{\mathrm{yawacc}} \end{array}].$$

In the augmented prediction step, $n_{x}$, should be the quantity of the augmented state $n_{x,\mathrm{aug}}$ with a size of 7, and λ also needs to be calculated as in Equation (15). Subsequently, the sigma points are predicted using Equation (16).

(15) $$\lambda \;=3-{\;n}_{x,\mathrm{aug}},$$

(16) $$X_{\mathrm{paukf},k,\mathrm{aug}}=\left(\mu_{\mathrm{paukf},k,\mathrm{aug}},{\;\mu }_{\mathrm{paukf},k,\mathrm{aug}}+\sqrt{\left(\lambda +n_{x,\mathrm{aug}}\right)P_{\mathrm{paukf},\;k,\mathrm{aug}}},{\;\mu }_{\mathrm{paukf},k,\mathrm{aug}}-\sqrt{\left(\lambda +n_{x,\mathrm{aug}}\right)P_{\mathrm{paukf},k,\mathrm{aug}}}\right).$$

The weight of each sigma point is calculated based on Equations (17) and (18). The predicted mean and covariance were calculated using Equations (19) and (20). The predicted value is the prior of the Bayesian distribution model. These predicted values should be updated when the measurement data are incoming.

(17) $$w_{\mathrm{paukf},i}=\frac{\lambda }{\lambda +n_{x,\mathrm{aug}}},\;\mathrm{when}\;i=0,$$

(18) $$w_{\mathrm{paukf},i}=\frac{1}{2\left(\lambda +n_{x,\mathrm{aug}}\right)},\;\mathrm{when}\;i=1\text{…}n_{x,\mathrm{aug}},$$

(19) $${\bar{x}}_{\mathrm{paukf},k+1|k}=\sum_{i=0}^{n_{a}}w_{\mathrm{paukf},i}X_{\mathrm{paukf},k+1|k,i},$$

(20) $${\bar{P}}_{k+1|k}=\sum_{i=0}^{2n_{a}}w_{\mathrm{paukf},i}(X_{\mathrm{paukf},k+1|k,i}-{\bar{x}}_{\mathrm{paukf},k+1|k}){\left(X_{\mathrm{paukf},k+1|k,i}-{\bar{x}}_{\mathrm{paukf},k+1|k}\right)}^{T}.$$

After predicting the new mean and covariance matrix based on the augmented sigma point, the algorithm no longer needs to consider noise as the acceleration noise information is already included in the state. Therefore, the augmented state changes back to a normal state with a size of 5. Until now, the prior probability was calculated based on sigma points. When using a Bayesian filter, measurement prediction can be implemented. Instead of using the original range sensor with noise from the vehicle, ${\hat{x}}_{\mathrm{PF}}$ is used in this step. This means that ${\hat{x}}_{\mathrm{PF}}$ becomes a virtual sensor, which is more precise than the original sensor. Since ${\hat{x}}_{\mathrm{PF}}$ already includes sensor information based on the particle filter, it optimally provides a more precise belief of the state. The measurement vector of the sensor is shown in Equation (21).

(21) $$z\;=\left[\begin{array}{c} x\\ y\\ \theta \end{array}\right].$$

The measurement model is shown in Equations (22) and (23). The particle filter measurement provides x, y, and yaw data. This means that the number of rows in matrix A is 3. Because the augmented state information is included in the state, the state of the UKF recovers to 5. This means that the number of columns in matrix A is 5.

(22) $$Z_{\mathrm{paukf},k+1|k,i}={\;\mathrm{AX}}_{\mathrm{paukf},k+1|k,i}+{\;\omega }_{\mathrm{paukf},k+1},$$

(23) $$A\;=\left[\begin{array}{ccccc} 1 & 0 & 0 & 0 & 0\\ 0 & 1 & 0 & 0 & 0\\ 0 & 0 & 0 & 1 & 0 \end{array}\right].$$

The predicted measurement mean is calculated based on the weight of each measurement’s sigma points, as shown in Equation (24).

(24) $${\bar{z}}_{\mathrm{paukf},k+1|k}=\sum_{i=1}^{n_{x}}w_{\mathrm{paukf},i}Z_{\mathrm{paukf},k+1|k,i}.$$

The predicted measurement covariance is calculated using Equation (25). R is the measurement noise covariance, as shown in Equation (26). The covariance is tuned according to the particle filter estimation results.

(25) $$S_{k+1|k}=\sum_{i=0}^{2n_{x}}w_{\mathrm{paukf},i}\left(Z_{\mathrm{paukf},k+1|k,i}-{\bar{z}}_{\mathrm{paukf},k+1|k}\right){\left(Z_{\mathrm{paukf},k+1|k,i}-{\;z\;¯}_{\mathrm{paukf},k+1|k}\right)}^{T}+\;R,$$

(26) $$R\;=\left[\begin{array}{ccc} \sigma_{x_{\mathrm{PF}}}{}^{2} & 0 & 0\\ 0 & \sigma_{y_{\mathrm{PF}}}{}^{2} & 0\\ 0 & 0 & \sigma_{\theta_{\mathrm{PF}}}{}^{2} \end{array}\right].$$

At this time, a measurement value is needed to calculate the posterior probability. The update step is similar to that of the Kalman filter. The only difference is that the UKF needs to calculate the cross-correlation value, according to Equation (27), between the sigma points in the state space and the measurement space.

(27) $$T_{k+1|k}=\sum_{i=0}^{2n_{x}}w_{\mathrm{paukf},i}\left(X_{\mathrm{paukf},k+1|k,i}-{\;x}_{\mathrm{paukf},k+1|k}\right){\left(Z_{\mathrm{paukf},k+1|k,i}-{\;z}_{\mathrm{paukf},k+1|k}\right)}^{T}.$$

Based on the cross-correlation matrix and the measurement covariance, the Kalman gain is then calculated as

(28) $$K_{k+1|k}={\;T}_{k+1|k}S_{k+1|k}^{-1}.$$

The state is updated using the measurement value ${\hat{x}}_{\mathrm{PF}}$, which is obtained from the particle filter estimation as

(29) $${\hat{x}}_{\mathrm{paukf}}={\;x}_{k+1|k+1}={\;x}_{k+1|k}+{\;K}_{k+1|k}\left({\hat{x}}_{\mathrm{PF}}-{\;z}_{k+1|k}\right).$$

The covariance matrix is then updated based on the updated Kalman gain and the measurement covariance matrix as

(30) $${\hat{P}}_{\mathrm{paukf}}={\;P}_{k+1|k+1}={\bar{P}}_{k+1|k}-{\;K}_{k+1|k}S_{k+1|k}K_{k+1|k}^{T}.$$

The terms $x_{k+1|k+1}$ and $P_{k+1|k+1}$ are the final estimation results of the PAUKF, which combines the bicycle model, CTRV motion model, Monte Carlo-based estimation, and unscented Kalman filter-based estimation. The complete pseudo code of the PAUKF algorithm is shown in Table 2.

## 3. Simulation Environment

The simulation of the PAUKF algorithm was performed using MATLAB. The autonomous driving toolbox is used for constructing the infrastructure, road structure, and vehicle kinematic model. The update frequency of the GPS is 10 Hz, and the frequency of the range sensor and gyroscope is 100 Hz. The noise of the GPS and the range sensor is simulated using the ground truth data appended with Gaussian noise and non-Gaussian noise. Gaussian noise is generated by using the normrnd function in MATLAB, and non-Gaussian noise is generated using a sinusoidal function (we assume the noise affected by the sinusoidal function) and a random number generator, as shown in Table 3 [45,46]. The seed of the random number is set as 50, and the sample time is set as 0.01 s. The variances fed into the PAUKF should be carefully tuned when applied to specific cases. It should be noted that even if the random seed is the same, the random number is generated depending on the number of times that it has been called. This means that any change in parameter changes the result because the input value changes.

**Figure 3:**
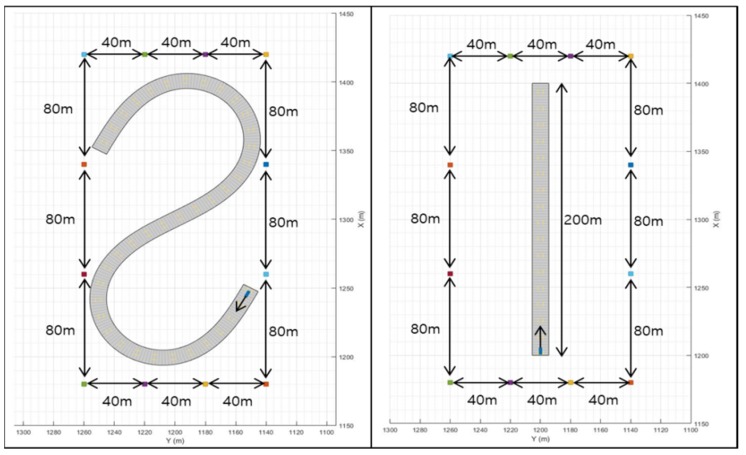
Simulation model.

The road simulated with three geometries is shown in Figure 3. There are S-shaped roads and a straight road in the X direction. An S-shaped road is used to verify the performance of each filter on a curved road. The straight-line road in the X direction is used in order to verify the performance of each filter on a straight road. There are 12 infrastructures around the road, and their positions are fixed, even when the map changes. In order to prevent the position of the infrastructures from affecting the performance of the filters, all the infrastructures are symmetrical. The velocity is set to a constant value, and the values are 60, 80, 100, and 120 km/h.

## 4. Analysis of Simulation Results

The simulation results are compared to evaluate the performance of the PAUKF. The evaluation parameter is based on the Root Mean Square Error (RMSE) as Equation (31) shows. We choose RMSE as an assessment parameter because the estimation performance of the filter can be compared intuitively by the numerical value of RMSE alone. In Equation (31), N indicates the number of data points. The trajectory of the estimated results and the ground truth of the vehicle’s trajectory are compared to verify the algorithm. The effect of the yaw angle is considered for both the x and y directions; therefore, there is no additional comparison of the yaw angle. The unit for all position parameters is meters.

(31) $$\left[\begin{array}{c} {\mathrm{RMSE}}_{\mathrm{est}}\\ {\mathrm{RMSE}}_{\mathrm{noise}} \end{array}\right]=\left[\begin{array}{c} \;\sqrt{[\sum_{i=1}^{N}{\left({\mathrm{Position}}_{{\mathrm{est}}_{i}}-{\mathrm{Position}}_{\mathrm{mean}\_{\mathrm{est}}_{i}}\right)}^{2}]/N}\\ \;\sqrt{[\sum_{i=1}^{N}{\left({\mathrm{Position}}_{{\mathrm{noise}}_{i}}-{\mathrm{Position}}_{\mathrm{mean}\_{\mathrm{noise}}_{i}}\right)}^{2}]/N} \end{array}\right].$$

### 4.1. Filter Performance on the S-Shaped Road

**Figure 4:**
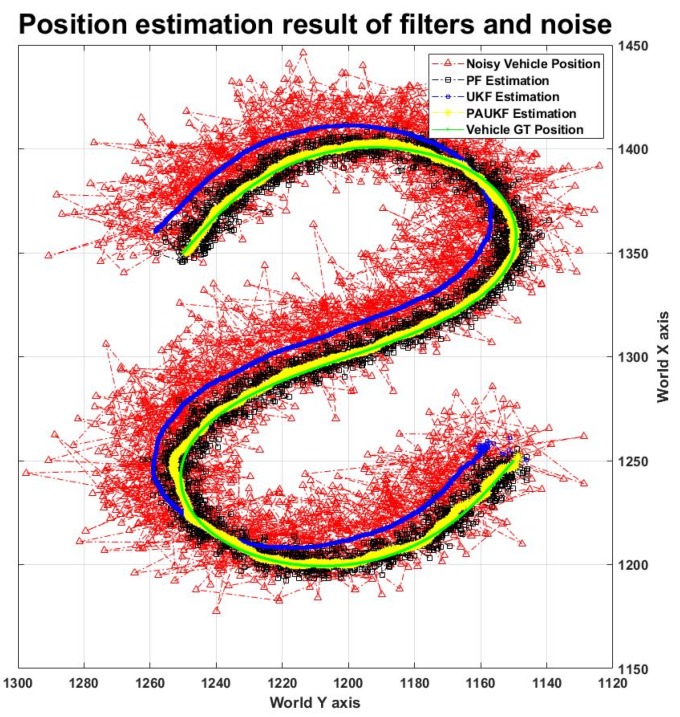
Position estimation result of the filters in the S curve road.

Figure 4 shows the trajectory results of the PF, UKF, and PAUKF, and noise in the S-shaped road. As the legend shows, the green line with a green circle is the ground truth trajectory, the dashed line with a red upward-pointing triangle is the noisy vehicle trajectory, the black dashed line with a black square is the PF estimated trajectory, the blue dashed line with a blue square is the UKF estimated trajectory, and the yellow dashed line with the yellow star marker is the PAUKF estimated trajectory. The data in Figure 4 are generated when the vehicle velocity is 60 km/h, and the noise is Gaussian, as shown in Table 3. The PF estimated trajectory is near the ground truth trajectory. However, the PF-estimated trajectory is not smooth, and the error is still large. This is because the PF localizes the vehicle position with noisy relative distance to each infrastructure and noisy vehicle data. Since there is no other measurement, it must be considered that the measurement is correct. Compared to that with the PF, the UKF-estimated trajectory is relatively smooth; however, it cannot filter the noise of the GPS data. Because the GPS measurement of the UKF has high variance and the UKF does not use range sensor data, the UKF believes the vehicle model more than the measurement. The noisy measurement also makes the UKF less sensitive to the changes in the position and yaw. Compared to that with the PF and UKF, the trajectory estimated by the PAUKF is more accurate and smoother. As it combines the smoothness of the UKF and the accuracy of the PF, the PAUKF reacts more quickly and precisely when the position and yaw change. Moreover, the PAUKF does not depend completely on either of the filters, trades off the filters, and generates even better results.

The filter performance results are shown in Table 4. Since the UKF does not use range sensor information, it is not appropriate to compare it with the PF and PAUF. Thus, there are no RMSEs for the UKF in Table 4. To compare with other literature, we calculate the mean value of estimation. The mean of estimation error for the PAUKF is 1.08 m and the variance is 0.7147 m, which is more precise than the mean of 1.69 m and variance of 1.63 m obtained by GANGNAM for similar noise [47]. In order to determine the performance of the filters in an extreme environment, the algorithm is tested under different velocity and noise environments. As mentioned in Section 3, even if the random seed is the same, the random number still changes depending on the number of times it has been called. Therefore, we analyzed the trend of every filter. It can be observed that the PF and PAUKF estimation errors increase slightly when the velocity increases. However, if we consider the magnitude of the RMSE of the changes in noise from 21.336 to 21.712 m, it can be found that the RMSE of the estimation error does not change even when the velocity increases from 60 to 120 km/h. Compared to the Gaussian noise, the non-Gaussian noise generated a larger mean value. Even so, the precision of the PF does not change even when the noise increases, and the precision is almost the same as the RMSE range of 5.489–5.959 m. The PAUKF has an RMSE range of 1.440–1.772 m, even when the noise increases and velocity increases. This is because PAUKF takes the PF estimation results as input and trades off the measurement and predicted value from the UKF. The trade-off is done using the cross-correlation function in Equation (27). Therefore, the PAUKF combines the recursiveness of the UKF and the location information of the infrastructures based on the PF. The PAUKF improves the accuracy by 4.028–4.049 m compared to the PF.

### 4.2. Filter Performance on a Straight Road in the X Direction

**Figure 5:**
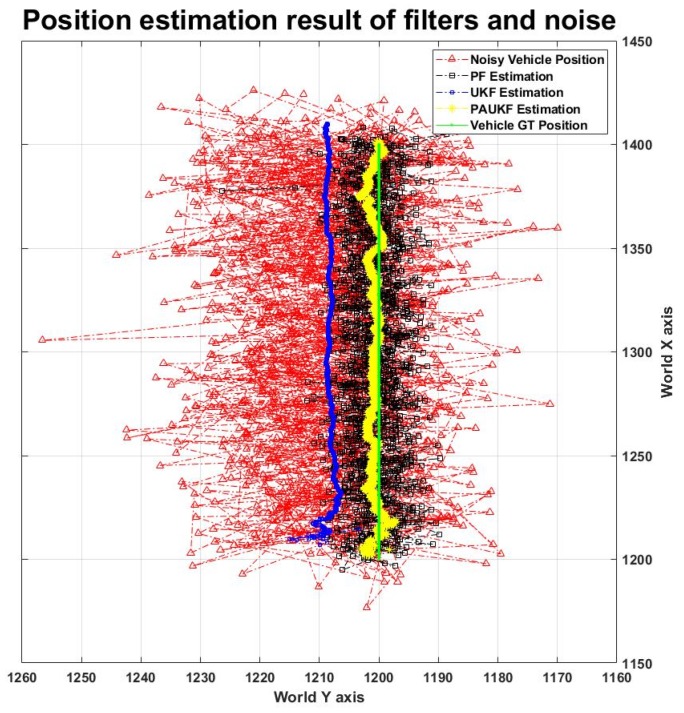
Position estimation result of the filters.

Figure 5 shows the trajectory results of the PF, UKF, and PAUKF, and the noise on a straight road in the X direction. The data in Figure 5 are generated when the vehicle velocity is 60 km/h, and the noise is Gaussian. The algorithm for a straight line is used to determine the performance when the vehicle only moves in the X direction. From Figure 5, it can be seen that the PAUKF converges to the ground truth better than the PF and UKF. Because the vehicle only moves in the X direction, there is no information about the movement in the Y direction. Therefore, even though the PAUKF estimation is better than that of the UKF, in order to improve the response time, the PAUKF tends to believe more about noise in the Y direction. As a result, the PAUKF is not sufficiently precise in the Y direction, as Figure 5 shows.

The results for filter performance are shown in Table 5 when the vehicle moves in the X direction. It can be found that the PF and PAUKF estimation properties are the same as those of the vehicle when it runs on an S-shaped road. The estimation results show that the performance of the algorithm does not change even when the map changes. The RMSE of the PF is 5.384–5.692 m and the PAUKF has an RMSE of 1.312–1.800 m even when the noise increases and the velocity increases. The PAUKF improves the accuracy by 3.892–4.072 m compared to the PF.

## 5. Conclusions

In this work, we propose a novel approach for a vehicle estimation algorithm, called the PAUKF, which combines the advantages of the PF and the UKF. The PAUKF combines the unscented transform property of a UKF with a sample-based PF to handle the localization problem in a bad GPS environment by using the range sensor and ground truth data of the infrastructure in an HD map. Owing to properties of the UKF, the PAUKF becomes more robust and precise compared to the original PF, given the same quantity of particles. The performance of the algorithm is stable and accurate (minimum RMSE: 1.44 m) when vehicles move along an S curve or any straight road at speeds of 60 to 120 km/h. The results of the simulation showed that the PAUKF has a significantly higher precision and stability than the PF and in previous research. In future work, we will try to implement the PAUKF in a real vehicle and incorporate the 3D range sensor data to upgrade the algorithm in the real world.

## Figures and Tables

**Table 1:** Pseudo code of the particle filter.

Order	Process
1	Start one sample time iteration
2	Initialization $X_{1,2\ldots N}$ particles
3	For 1 to N do
4	${\bar{X}}_{k+1}=\;\mathrm{prediction}\;\mathrm{model}\left(X_{k},{\;u}_{t}\right)$
5	End for
6	$Z_{k+1}=\;\mathrm{measurent}\;\mathrm{input}$
7	$w_{\left[1,2\ldots N\right]}=\;\mathrm{multivariable}\;\mathrm{normal}\;\mathrm{distribution}\left({\bar{X}}_{k+1},{\;Z}_{k+1},{\;\sigma }_{\mathrm{distance}},{\;\sigma }_{\mathrm{orientation}}\right){\bar{X}}_{k+1}$
8	Return ${\hat{X}}_{\mathrm{PF}}=\;f\left(X_{k+1}^{\left[1,2\ldots N\right]},{\;w}_{\left[1,2\ldots N\right]}\right)$
9	End one sample time iteration

**Table 2:** Pseudocode of the particle-aided unscented Kalman filter (PAUKF).

Order	Process
1	Start one sample time iteration
2	Initialization $X_{1,2\ldots N}$ particles
3	For 1 to N do
4	${\bar{X}}_{k+1}=\;\mathrm{prediction}\;\mathrm{model}\left(X_{k},{\;u}_{t}\right)$
5	End for
6	${\hat{x}}_{\mathrm{PF}}=\;f\left({\bar{X}}_{k+1},{\;w}_{\left[1,2\ldots N\right]}\right)$
7	For 1 to $n_{\mathrm{aug}}$ do
8	$X_{k+1}$ = unscented transform ($\lambda ,{\;x}_{k},\sigma )$
9	${\bar{x}}_{\mathrm{paukf},k+1|k}$ = CTRV model(based state prediction
10	${\bar{z}}_{\mathrm{paukf},k+1|k}$ = A(${\bar{x}}_{\mathrm{paukf},k+1|k}$) for measurement prediction
11	${\hat{x}}_{\mathrm{paukf}},{\hat{P}}_{\mathrm{paukf}}=\;\mathrm{state}\;\mathrm{update}\left(T_{k+1|k},{\;S}_{k+1|k},{\bar{z}}_{\mathrm{paukf},k+1|k},{\;x\;¯}_{\mathrm{paukf},k+1|k},{\hat{x}}_{\mathrm{PF}},R\right)$
12	End one sample time iteration

**Table 3:** Simulated noisy environment setting.

Noise Name	Generate Method
GPS x error (Gaussian)	~N(9.65, 12.2) [46]
GPS x error (Non_Gaussian)	~15sin(N(0, 1))+N(9.65, 12.2)+5)
GPS y error (Gaussian)	~N(8.34, 12.33) [46]
GPS y error (Non_Gaussian)	~15sin(N(0, 1))+N(8.34, 12.33)+5)
Velocity error	~sin(N(0, 1))
Yaw error	~sin(N(0, 10))
Yaw rate error	~sin(N(0, 10))
Random seed	50

**Table 4:** Total Root Mean Square Error (RMSE) of filters in different conditions (unit: m).

	With Gaussian Noise			With Non-Gaussian Noise		
Velocity	Noise	PF	PAUKF	Noise	PF	PAUKF
60 km/h	21.336	5.634	1.451	29.796	5.959	1.655
80 km/h	21.310	5.600	1.651	29.730	5.579	1.440
100 km/h	21.154	5.720	1.501	29.430	5.631	1.616
120 km/h	21.712	5.800	1.772	29.934	5.489	1.454

**Table 5:** RMSE of filters in different conditions (unit: m).

	With Gaussian Noise			With Non-Gaussian Noise		
Velocity	Noise	PF	PAUKF	Noise	PF	PAUKF
60 km/h	21.411	5.655	1.486	29.376	5.658	1.659
80 km/h	21.518	5.546	1.312	28.987	5.491	1.526
100 km/h	21.848	5.692	1.800	29.274	5.615	1.482
120 km/h	21.363	5.383	1.710	30.078	5.617	1.719

## Nomenclature

bel(x)	Belief of state
$x_{\left[1,2,3,\ldots i\right]}$	States of particle1, Particle 2, … Particle i
$w_{\left[1,2,3,\ldots i\right]}$	Weights of particle1, particle 2, … particle i
${\bar{X}}_{k+1}$	State at sample time k + 1
K	Timestamp
$x_{k}$, $y_{k}$	Vehicle position in the x, y dimension at time k
$v_{k}$	Vehicles position in the x dimension at time k
$\theta_{k}$	Yaw angle at time k
$\dot{\theta_{k}\left(\mathrm{dt}\right)}$, $\dot{\theta }$	Yaw rate of vehicle at time k
Δt, dt	Sample time
$Z_{k+1}$	Measurement vector at time k + 1
$d_{\left[i\right]}$	Distance of ego vehicle to ith beacon
$\Delta \theta_{\left[i\right]}$	Relative angle of vehicle orientation and ith beacon
$x_{b,i}$, $y_{b,i}$	Relative distance of vehicle and ith beacon
$\epsilon_{d}$	Noise distance measurement
$\epsilon_{\Delta \theta }$	Noise of angle measurement
$p\left(x_{i},y_{i}\right)$	Multivariable normal distribution
$\sigma_{x}$, $\sigma_{y}$	Covariance of sensor range noise in the x- and y-directions
$x_{\mathrm{paukf},\;k,\mathrm{aug}}$	State of PAUKF
$w_{\mathrm{velacc}}$	Noise of vehicle acceleration
$w_{\mathrm{yawacc}}$	Noise of vehicle yaw acceleration
$\sigma_{\mathrm{velacc}}$	Variance of noise of vehicle acceleration
$\sigma_{\mathrm{velacc}}$	Variance of noise in vehicle yaw acceleration
$P_{k,\mathrm{aug}}$	Variance matrix of PAUKF.
$X_{\mathrm{paukf},\;k+1,\mathrm{aug}}$	Augmented state with sigma points of PAUKF at time k + 1
$\mu_{\mathrm{paukf},k,\mathrm{aug}}$	Mean value of augmented state of PAUKF at time k
$n_{x,\mathrm{aug}}$	Number of augmented states
$w_{\mathrm{paukf},i}$	Weight of ith sigma point
λ	Sigma point design parameter
${\bar{x}}_{\mathrm{paukf},k+1|k}$	Predicted state based on the weight of sigma points and states
${\bar{P}}_{k+1|k}$	Predicted variance based on sigma points and predicted state mean
$\omega_{\mathrm{paukf},k+1}$	Measurement noise of PAUKF.
$Z_{\mathrm{paukf},k+1|k,i}$	Measurement prediction based on sigma points.
$X_{\mathrm{paukf},k+1|k,i}$	Sigma points of state
A	Measurement transition model.
$z_{\mathrm{paukf},k+1|k}$	Predicted measurement based on sigma points and weights
$S_{k+1|k}$	Predicted measurement covariance matrix.
R	Variance matrix of the measurement noise.
$\sigma_{x_{\mathrm{pf}}}$	Covariance of PF estimation in the x dimension
$\sigma_{y_{\mathrm{pf}}}$	Covariance of PF estimation in the y-dimension
$T_{k+1|k}$	Cross-correlation matrix of PAUKF
$K_{k+1|k}$	Kalman gain of PAUKF
${\hat{x}}_{\mathrm{PAUFK}}$	Final state estimation of PAUKF.
${\hat{P}}_{\mathrm{PAUFK}}$	Final state variance matrix of PAUKF
